# Astegolimab for chronic obstructive pulmonary disease with frequent exacerbations: pooled analysis of the ALIENTO and ARNASA trials

**DOI:** 10.1093/ajrccm/aamag226

**Published:** 2026-05-18

**Authors:** Jadwiga A Wedzicha, Àlvar Agustí, Christopher E Brightling, Peter Calverley, James D Chalmers, Neil J Greening, MeiLan K Han, Divya Mohan, Julie Ng, Alberto Papi, Nicolas Roche, Rebecca Saenz, Katerina Samara, Ruth Tal-Singer, Claus F Vogelmeier, Xiaoying Yang, Bartolome R Celli

**Affiliations:** National Heart and Lung Institute, Imperial College London, London, United Kingdom; Department of Medicine, University of Barcelona, Barcelona, Spain; Respiratory Institute, Hospital Clinic Barcelona, Barcelona, Spain; FCRB-IDIBAPS, Barcelona, Spain; CIBER Enfermedades Respiratorias, Instituto de Salud Carlos III, Spain; Division of Respiratory Sciences, Institute for Lung Health, University of Leicester, Leicester, United Kingdom; Institute of Life Course and Medical Sciences, University of Liverpool, Liverpool, United Kingdom; Radcliffe Department of Medicine, University of Oxford, Oxford, United Kingdom; Division of Respiratory Sciences, Institute for Lung Health, University of Leicester, Leicester, United Kingdom; Division of Pulmonary and Critical Care, Department of Internal Medicine, University of Michigan, Ann Arbor, MI, United States; Product Development, Genentech, Inc, South San Francisco, CA, United States; Clinical Science, Early Clinical Development, Genentech, Inc, South San Francisco, CA, United States; Pulmonary Division, University of Ferrara, St Anna University Hospital, Ferrara, Italy; Pneumologie, Université Paris Cité, APHP Centre, Hôpital et Institut Cochin, Paris, France; Product Development, Genentech, Inc, South San Francisco, CA, United States; Roche Pharma Product Development, F. Hoffmann-La Roche, Ltd, Basel, Switzerland; Global Allergy & Airways Patient Platform, Vienna, Austria; Pulmonary, Critical Care and Sleep Medicine, Department of Medicine, Philipps University of Marburg, German Center for Lung Research, Marburg, Germany; Product Development Data Sciences, Genentech, Inc, South San Francisco, CA, United States; Pulmonary and Critical Care, Mass General Brigham Hospital, Harvard University Medical School, Boston, MA, United States

**Keywords:** inflammation, ST2 monoclonal antibody, interleukin-33

## Abstract

**Rationale:**

The efficacy and safety of astegolimab, an anti-ST2 monoclonal antibody, was evaluated in participants with chronic obstructive pulmonary disease (COPD) and frequent exacerbations in the pivotal ALIENTO and ARNASA trials.

**Objectives:**

To report the prespecified pooled analysis of ALIENTO and ARNASA.

**Methods:**

ALIENTO and ARNASA were randomized, double-blind, placebo-controlled trials with similar designs and included participants with COPD, a history of frequent exacerbations, and current/former smoking status, irrespective of blood eosinophil count and chronic bronchitis. Participants were randomized 1:1:1 to astegolimab 476 mg every 2 weeks (Q2W), astegolimab every 4 weeks (Q4W), or placebo for 52 weeks, plus optimized maintenance therapy. The primary endpoint was annualized rate of moderate/severe exacerbations. Secondary endpoints included annualized rate of severe exacerbations. A hierarchical statistical plan was followed with hypothesis testing of secondary efficacy endpoints gated on the primary efficacy endpoint success.

**Measurements and Main Results:**

A total of 2682 participants were included in the pooled intent-to-treat population. Astegolimab significantly reduced the annualized rate of moderate/severe exacerbations by 15% in the Q2W arm (adjusted rate ratio, 0.85 [95% CI, 0.76-0.96]; *P* = .0077) and by 12% in the Q4W arm (rate ratio, 0.88 [95% CI, 0.78-0.99]; *P* = .0265). ­A ­nominally significant reduction in the annualized rate of severe COPD exacerbations (rate ratio, 0.68 [95% CI, 0.52-0.87]; *P* = .0028) was observed for astegolimab Q2W vs placebo. Astegolimab was well tolerated.

**Conclusions:**

Astegolimab every 2 weeks reduced the annualized rate of moderate/severe exacerbations in a clinically heterogeneous population of participants with COPD and frequent exacerbations.

**Clinical trial registration:**

ClinicalTrials.gov: NCT05037929; NCT05595642

At a Glance Commentary **Scientific Knowledge on the Subject:**

Many patients with chronic obstructive pulmonary disease (COPD) continue to experience exacerbations despite receiving optimized maintenance therapy. Currently, add-on biologic therapies for uncontrolled COPD are approved for use only in patients with an eosinophilic phenotype. Astegolimab, a human anti-ST2 monoclonal antibody, blocks IL-33 activity and subsequent downstream neutrophilic and eosinophilic inflammatory pathways. The pivotal phase 2b ALIENTO and phase 3 ARNASA trials evaluated the efficacy and safety of astegolimab in participants with COPD and frequent exacerbations, enrolled regardless of eosinophil count or chronic bronchitis or current/former smoking status. Both trials reported consistent data across multiple endpoints with astegolimab every 2 weeks (Q2W) vs placebo, including a 15% reduction in annualized rate of moderate/severe exacerbations, despite only ALIENTO achieving statistical significance.

**What This Study Adds to the Field:**

In this prespecified pooled analysis of ALIENTO and ARNASA, astegolimab Q2W vs placebo significantly reduced the annualized rate of moderate/severe exacerbations. A nominally significant reduction in severe exacerbations was also observed and a responder analysis of St George’s Respiratory Questionnaire for COPD suggested a clinically meaningful improvement in health-related quality of life with astegolimab Q2W vs placebo. Astegolimab was well tolerated. The results of this pooled analysis further support the efficacy of astegolimab Q2W in a clinically heterogeneous population of patients with COPD and frequent exacerbations.

## Introduction

Chronic obstructive pulmonary disease (COPD) is a heterogeneous lung condition associated with chronic respiratory symptoms resulting from abnormalities of the airways and/or alveoli, leading to persistent, often progressive, airflow obstruction.^1^ Despite receiving optimized inhaled pharmacological therapy, many patients continue to experience exacerbations,^1-3^ which are associated with increased morbidity, mortality, healthcare resource utilization, and reduced quality of life.^2-5^ The consequences of severe exacerbations (ie, those requiring hospitalization) are substantial; for example, the risk of a cardiovascular event following a severe exacerbation is around double that following a moderate exacerbation.^6^ Every moderate or severe exacerbation cumulatively increases the risk of subsequent exacerbations and death, with a greater risk following severe exacerbations.^3^^,^^7^

When exposed to harmful stimuli such as viruses, bacteria, or cigarette smoke, epithelial cells release alarmins such as interleukin-33 (IL-33).^8^^,^^9^ IL-33 binding to its receptor ST2 triggers downstream pathways that drive neutrophilic (type 1/3) and eosinophilic (type 2) inflammation.^9-11^ These inflammatory responses are important contributors to the initiation, maintenance, and progression of exacerbations via persistent inflammation and lung tissue remodeling.^9^^,^^12^

Astegolimab is a human anti-ST2 IgG2 monoclonal antibody that blocks IL-33 activity and subsequent inflammatory responses.^9^^,^^13^ The efficacy and safety of astegolimab was evaluated in participants with COPD and a history of frequent exacerbations in the phase 2b ALIENTO (NCT05037929) and phase 3 ARNASA (NCT05595642) pivotal trials.^14^

In ALIENTO, astegolimab every 2 weeks (Q2W) was associated with a lower annual rate of moderate or severe exacerbations vs placebo. The effect size of 15% was consistent in both studies but did not meet statistical significance in ARNASA.^14^ Astegolimab was well tolerated, and the safety profile was consistent with previous trials.^14^

Pooling data from multiple trials provides a more robust estimate of the magnitude of effect in a broad patient population and increases the statistical power of subgroup analyses, in particular where the results of the pooled analyses are directionally consistent within each of the individual studies. Here we report the results of the prespecified pooled analysis of the ALIENTO and ARNASA trials. Some of the results of this analysis have been previously reported in an abstract.^15^

## Methods

### Trial design

The detailed trial designs and eligibility criteria have been described previously.^14^ In brief, participants were enrolled across a total of 39 countries and randomized 1:1:1 to receive astegolimab 476 mg Q2W or every 4 weeks (Q4W) or placebo, added to optimized maintenance therapy for 52 weeks. The trials complied with the International Council for Harmonisation E6 guideline for Good Clinical Practice and the principles of the Declaration of Helsinki and were approved by the relevant institutional review boards and ethics committees. Written informed consent was obtained from participants and safety was monitored by an independent data monitoring committee.

### Participants

Participants were aged 40 to 90 years (ALIENTO) or 40 to 80 years (ARNASA), had a COPD diagnosis for >12 months with a history of frequent exacerbations (≥2 moderate or severe exacerbations within a 12-month period in the 24 months prior to screening [ALIENTO] or within the 12 months prior to screening [ARNASA]), had current/former smoking status, and had post-bronchodilator percent predicted forced expiratory volume in 1 second (FEV_1_) ≥20 and <80% and FEV_1_/forced vital capacity <0.70 at screening. Participants were receiving optimized maintenance therapy (inhaled corticosteroid [ICS] + long-acting muscarinic antagonist [LAMA] + long-acting β-agonist [LABA], ICS + LABA, or LAMA + LABA) and were enrolled irrespective of blood eosinophil count (BEC) or chronic bronchitis.

### Pooled analysis outcomes

This analysis is based on the primary analysis for ALIENTO and ARNASA. The primary endpoint was the annualized rate of moderate or severe COPD exacerbations over 52 weeks. Moderate exacerbations were defined as new/increased COPD symptoms that led to treatment (≥3 days) with systemic corticosteroids (>10 mg/day prednisolone equivalent) and/or antibiotics, and severe exacerbations as new or increased COPD symptoms leading to hospitalization for ≥24 hours or death. Secondary efficacy endpoints were annualized rate of severe exacerbations, annualized rate of moderate or severe exacerbations in participants with baseline BEC <300 cells/μL, time to first moderate or severe exacerbation, proportion of participants with decrease from baseline of ≥4 points in St George’s Respiratory Questionnaire for COPD (SGRQ-C),^16^ absolute change from baseline in SGRQ-C, absolute change from baseline in Evaluating Respiratory Symptoms in COPD (E-RS:COPD) total score, and absolute change from baseline in post-bronchodilator FEV_1_ (see Table S1 for hierarchical testing order). Safety was assessed by incidences of treatment-emergent adverse events (AEs) including infections and major adverse cardiovascular events (MACEs) (see Supplementary Methods), laboratory tests, vital signs, and electrocardiograms.

### Statistical analysis

The statistical analysis plan (SAP) for the pooled analysis was prespecified (the final amendment to the pooled SAP was made in May 2025, prior to ARNASA database lock) and based on the ARNASA SAP.^14^ Efficacy was evaluated in the modified intent-to-treat population, which comprised participants who received ≥1 dose of trial drug, based on assignment at randomization. Hypothesis testing of secondary efficacy endpoints was gated on the success of the primary efficacy endpoint, using fixed sequence testing procedures (see Table S1 for hierarchical testing order). Safety was evaluated in the safety evaluable population, which comprised participants who received ≥1 dose of trial drug, based on treatment received. AEs were adjusted for patient-years at risk. See Supplementary Methods for further details.

## Results

### Participants

Overall, 2682 participants were randomized across both trials and included in the pooled intent-to-treat population (1302 from ALIENTO, 1380 from ARNASA). A total of 2676 participants received at least one dose of trial treatment (astegolimab Q2W, *n* = 892; astegolimab Q4W, *n* = 896; placebo, *n* = 888), and of those, 415 (15.5%) discontinued trial treatment prior to week 52. The trial dropout rate was 13.8% and balanced across treatment arms. Treatment compliance rates were high, with 87.6% of participants receiving 19 to 26 treatment doses (astegolimab or placebo). See Figure S1 for full participant disposition. The baseline characteristics of participants were generally balanced across treatment arms and representative of a clinically heterogeneous COPD population (Table 1, Table S2); 60.3% of the population was male, 76.8% were receiving triple inhaled therapy (LABA/LAMA/ICS) at baseline, and 87.2% had baseline BEC <300 cells/µL.

**Table 1:** Baseline demographics and characteristics of astegolimab every 2 weeks and placebo arms (modified intent-to-treat population).

Characteristic	Astegolimab Q2W	Placebo
	**(*n*** = **892)**	**(*n*** = **888)**
**Age, y, mean (SD)**	66.76 (7.93)	67.14 (7.27)
**Sex, No. (%)**		
** Male**	538 (60.3)	536 (60.4)
** Female**	354 (39.7)	352 (39.6)
**Race, No. (%)**		
** American Indian or Alaska Native**	9 (1.0)	11 (1.2)
** Asian**	112 (12.6)	123 (13.9)
** Black or African American**	29 (3.3)	35 (3.9)
** Native Hawaiian or other Pacific Islander**	5 (0.6)	2 (0.2)
** White**	716 (80.3)	699 (78.7)
** Multiple**	8 (0.9)	10 (1.1)
** Not reported or unknown^a^**	13 (1.5)	8 (0.9)
**Ethnicity, No. (%)**		
** Hispanic or Latino**	147 (16.5)	154 (17.3)
**Smoking status, No. (%)**		
** Current**	302 (33.9)	303 (34.1)
** Former**	590 (66.1)	585 (65.9)
**Blood eosinophil counts**		
** No.**	888	888
** Mean (SD), cells/μL**	183.5 (159.1)	176.5 (116.2)
** <150 cells/μL, No. (%)**	459 (51.7)	427 (48.1)
** ≥150 to <300 cells/μL, No. (%)**	306 (34.5)	360 (40.5)
** ≥300 cells/μL, No. (%)**	123 (13.9)	101 (11.4)
**FEV_1_**		
** No.**	889	884
** Mean FEV_1_ (SD)** ^b^ **, L**	1.23 (0.48)	1.21 (0.50)
** Mean %FEV_1_ (SD)** ^b^ **, %**	45.8 (15.2)	45.2 (15.6)
**Number of COPD exacerbation within 12 months prior to screening** ^c^ **, No. (%)**		
** No.**	891	886
** 2**	612 (68.7)	649 (73.3)
** >2**	279 (31.3)	237 (26.7)
**COPD standard of care at baseline^d^, No. (%)**		
** No.**	891	887
** ICS/LABA/LAMA**	695 (78.0)	672 (75.8)
** ICS/LABA**	128 (14.4)	133 (15.0)
** LABA/LAMA**	67 (7.5)	78 (8.8)
** ICS**	1 (0.1)	2 (0.2)
** ICS/LAMA**	0 (0)	1 (0.1)
** LAMA**	0 (0)	1 (0.1)
**Use of azithromycin^d^, No. (%)**	15 (1.7)	38 (4.3)
**Use of roflumilast^d^, No. (%)**	35 (3.9)	26 (2.9)
**Diagnosis of chronic bronchitis, No. (%)**	420 (47.1)	427 (48.1)
**GOLD stage at baseline, No. (%)**		
** No.**	889	884
** GOLD 1 (mild)**	9 (1.0)	11 (1.2)
** GOLD 2 (moderate)**	333 (37.5)	303 (34.3)
** GOLD 3 (severe)**	392 (44.1)	408 (46.2)
** GOLD 4 (very severe)**	155 (17.4)	162 (18.3)
**mMRC, No. (%)**		
** 0**	24 (2.7)	32 (3.6)
** 1**	58 (6.5)	63 (7.1)
** 2**	419 (47.0)	392 (44.1)
** 3**	372 (41.7)	378 (42.6)
** 4**	19 (2.1)	23 (2.6)
**Mean SGRQ-C total score (SD)**	59.9 (17.7) [*n* = 886]	59.6 (18.2) [*n* = 881]
**Mean E-RS:COPD total score (SD)**	16.0 (6.5) [*n* = 872]	15.9 (6.6) [*n* = 865]

Abbreviations: %FEV_1_, percent predicted forced expiratory volume in 1 second; COPD, chronic obstructive pulmonary disease; E-RS:COPD, Evaluating Respiratory Symptoms in COPD; FEV_1_, forced expiratory volume in 1 second; GOLD, Global Initiative for Chronic Obstructive Lung Disease; ICS, inhaled corticosteroid; LABA, long-acting β-agonist; LAMA, long-acting muscarinic antagonist; mMRC, modified Medical Research Council; Q2W, every 2 weeks; SD, standard deviation; SGRQ-C, St George’s Respiratory Questionnaire for COPD.

a Unknown refers to participants for whom race is not known. Participants who refuse to answer or do not identify with any of the options listed are also counted under unknown. Not reported refers to participants for whom race cannot be collected per local laws and regulations.

b All spirometry parameters are based on post-bronchodilator sessions.

c Number of COPD exacerbation within 12 months prior to screening was evaluated in a 12-month period in the 24 months prior to screening in ALIENTO.

d Baseline use of COPD standard of care, azithromycin, and roflumilast is determined based on prior and concomitant drugs that were initiated before the initial study drug and were ongoing at the time of the initial study drug administration.

### Primary endpoint

**Figure 1:**
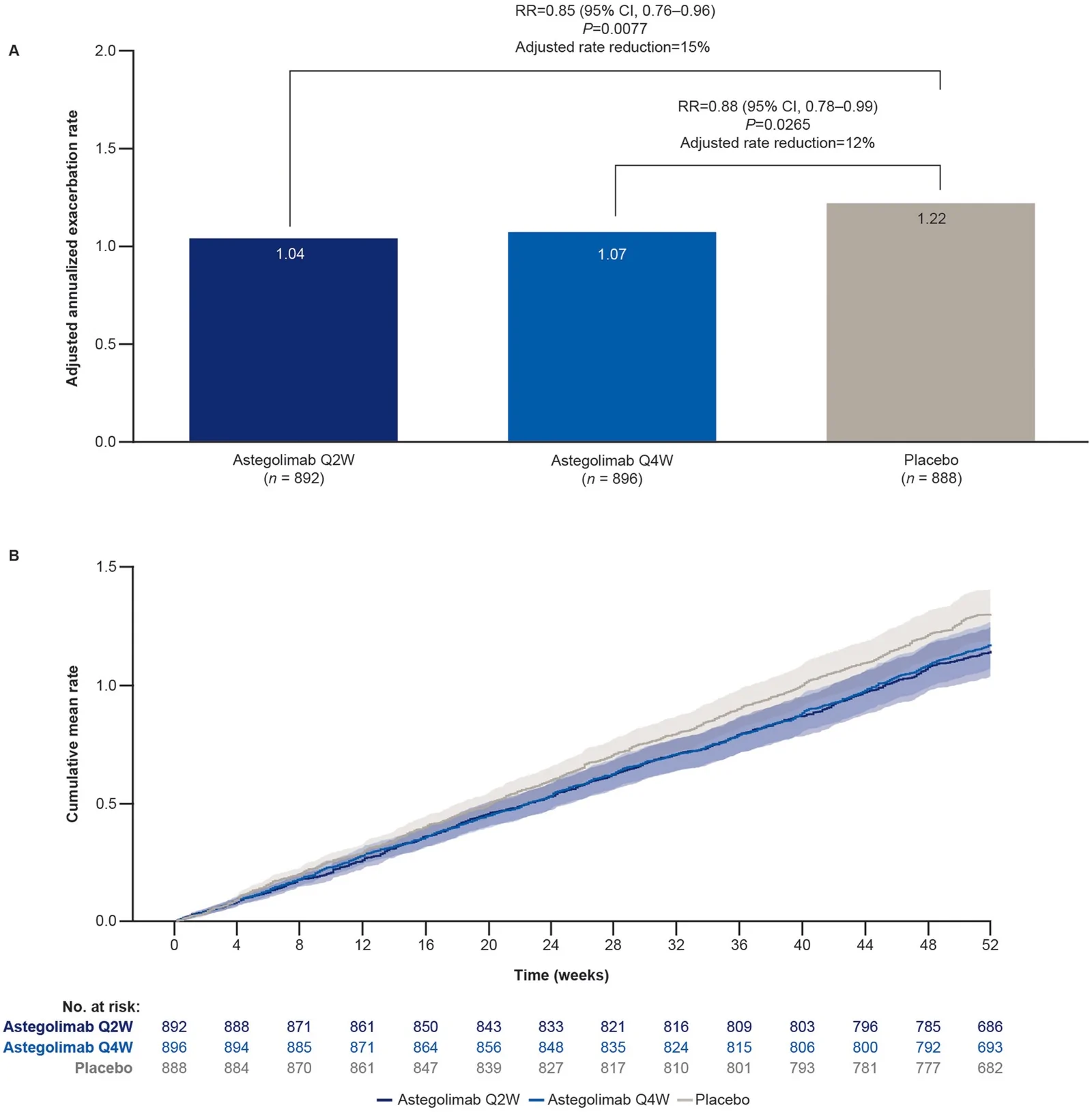
Primary endpoint: (A) adjusted annualized rate of moderate or severe COPD exacerbations over the 52-week treatment period for astegolimab Q2W vs placebo; (B) cumulative mean rate of moderate or severe COPD exacerbations for astegolimab Q2W and Q4W vs placebo (mITT population). Abbreviations: CI, confidence interval; COPD, chronic obstructive pulmonary disease; mITT, modified intent-to-treat; Q2W, every 2 weeks; Q4W, every 4 weeks; RR, rate ratio.

The adjusted annualized rates of moderate or severe exacerbations were 1.04 for astegolimab Q2W, 1.07 for astegolimab Q4W, and 1.22 for placebo. Astegolimab Q2W significantly reduced the adjusted annualized rate of moderate or severe exacerbations by 15% vs placebo (rate ratio [RR], 0.85 [95% CI, 0.76-0.96]; *P* = .0077; Figure 1). Compared with placebo, astegolimab Q4W also significantly reduced the adjusted annualized rate of moderate or severe exacerbations by 12% (RR, 0.88 [95% CI, 0.78-0.99]; *P* = .0265; Figure 1).

**Figure 2:**
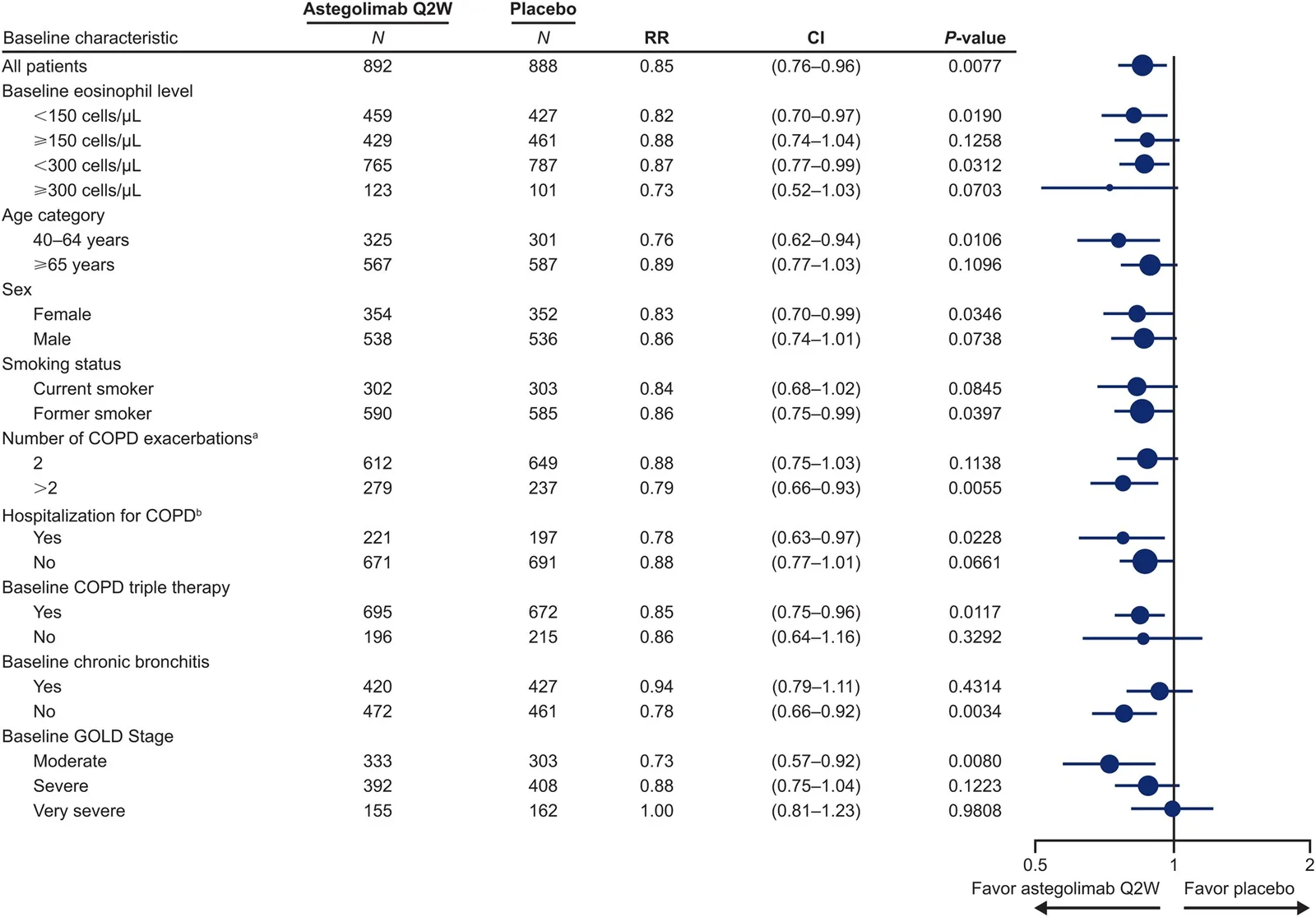
Adjusted annualized rate of moderate or severe COPD exacerbations for astegolimab Q2W vs placebo, by selected prespecified subgroups (mITT population). ^a^For ALIENTO, the number of COPD exacerbations was based on those occurring in a 12-month period within the 24 months prior to screening. For ARNASA, it was based on 12 months prior to screening. ^b^Hospitalization for COPD for >24 hours within the last 12 months prior to screening. Abbreviations: CI, confidence interval; COPD, chronic obstructive pulmonary disease; GOLD, Global Initiative for Chronic Obstructive Lung Disease; mITT, modified intent-to-treat; Q2W, every 2 weeks; RR, rate ratio.

Astegolimab Q2W reduced exacerbation frequency across prespecified subgroups, including current/former smoking status and baseline BEC (Figure 2). When split by a BEC cutoff of 150 cells/μL, participants with <150 cells/μL had an 18% reduction in exacerbation frequency for astegolimab Q2W vs placebo (RR, 0.82 [95% CI, 0.70-0.97]; *P* = .0190), and those with ≥150 cells/μL had a 12% reduction (RR, 0.88 [95% CI, 0.74-1.04]; *P* = .1258). When split by a BEC cutoff of 300 cells/μL, participants with <300 cells/μL had a 13% reduction in exacerbation frequency for astegolimab Q2W vs placebo (RR, 0.87 [95% CI, 0.77-0.99]; *P* = .0312; the <300 cells/μL subgroup is also a secondary endpoint [Table 2]) and those with ≥300 cells/μL had a 27% reduction (RR, 0.73 [95% CI, 0.52-1.03]; *P* = .0703). Annualized exacerbation rate reduction analysis with astegolimab Q2W and Q4W showed similar responses across mutually exclusive eosinophil subgroups (<150 cells/μL, ≥150 to <300 cells/μL, and ≥300 cells/μL), with overlapping CIs (Figure S2).

**Table 2:** Secondary efficacy endpoints for astegolimab every 2 weeks vs placebo (modified intent-to-treat population).

Endpoint	Astegolimab Q2W	Placebo
	**(*n*** = **892)**	**(*n*** = **888)**
**Absolute change from baseline in SGRQ-C at week 52**		
** Adjusted mean (SE)**	−7.11 (0.59) [*n* = 734]	−5.80 (0.59) [*n* = 741]
** Difference in adjusted mean vs placebo (95% CI)**	−1.31 (−2.95 to 0.34) *P* = .1195	…
**Annualized rate of severe COPD exacerbations over the 52-week treatment period**		
** Total number of severe exacerbation events**	171	220
** Adjusted rate**	0.15	0.22
** Adjusted rate ratio vs placebo (95% CI)**	0.68 (0.52-0.87) *P* = .0028^a^	…
**Time to first moderate or severe COPD exacerbation during the 52-week treatment period**		
** Median (95% CI), weeks**	45.0 (38.1–NE)	36.3 (32.9-44.4)
** Stratified hazard ratio vs placebo (95% CI)**	0.89 (0.79-1.01) *P* = .0779	…
**Absolute change from baseline in post-bronchodilator FEV_1_ at week 52**		
** Adjusted mean (SE), mL**	−4.47 (8.13) [*n* = 706]	−19.48 (8.12) [*n* = 710]
** Difference in adjusted mean vs placebo (95% CI)**	15.01 (−7.52 to 37.53) *P* = .1916	…
**Absolute change from baseline in E-RS:COPD total score at week 52**		
** Adjusted mean (SE)**	−1.77 (0.21) [*n* = 685]	−1.68 (0.21) [*n* = 697]
** Difference in adjusted mean vs placebo (95% CI)**	−0.09 (−0.66 to 0.48) *P* = .7598	…
**Proportion of patients with decrease from baseline of ≥4 points in SGRQ-C at week 52**		
** Adjusted responder rate, %**	56.6 [*n* = 852]	49.2 [*n* = 845]
** Odds ratio vs placebo (95% CI)**	1.35 (1.11-1.64) *P* = .0029^a^	…
**Annualized rate of moderate or severe COPD exacerbations over the 52-week treatment period in patients with baseline eosinophil count <300 cells/μL**		
** Adjusted rate**	1.04 [*n* = 765]	1.19 [*n* = 787]
** Adjusted rate ratio vs placebo (95% CI)**	0.87 (0.77-0.99) *P* = .0312^a^	…

Endpoints listed in order of fixed sequence testing procedures.

Abbreviations: CI, confidence interval; COPD, chronic obstructive pulmonary disease; E-RS:COPD, Evaluating Respiratory Symptoms in COPD; FEV_1_, forced expiratory volume in 1 second; NE, not estimable; Q2W, every 2 weeks; SE, standard error; SGRQ-C, St George’s Respiratory Questionnaire for COPD.

a Nominally significant due to an earlier break in the testing hierarchy.

Prespecified subgroups which suggested an enhanced treatment benefit of astegolimab Q2W vs placebo in both individual trials as well as the pooled analysis were participants with a history of >2 exacerbations (21% reduction; RR, 0.79 [95% CI, 0.66-0.93]; *P* = .0055), those with moderate airflow obstruction (Global Initiative for Chronic Obstructive Lung Disease [GOLD] stage 2: 27% reduction; RR, 0.73 [95% CI, 0.57-0.92]; *P* = .0080), and those without chronic bronchitis (22% reduction; RR, 0.78 [95% CI, 0.66-0.92]; *P* = .0034), while in the astegolimab Q4W arm only participants with a history of >2 exacerbations met these criteria (Figure S3).

### Secondary endpoints

Secondary efficacy endpoints for astegolimab Q2W vs placebo are summarized in Table 2. The statistical hierarchy was broken at the analysis of absolute change from baseline in SGRQ-C at week 52, meaning statistical significance cannot be claimed for any secondary endpoints. Astegolimab Q4W secondary endpoint data are provided in Table S3 but are not discussed further here.

#### Severe exacerbations

A 32% reduction in the annualized rate of severe COPD exacerbations was observed for astegolimab Q2W vs placebo (RR, 0.68 [95% CI, 0.52-0.87]; *P* = .0028; Table 2). This result was nominally significant due to the break in testing hierarchy (Table 2, Table S1). Overall, participants in the astegolimab Q2W arm experienced a total of 171 severe exacerbations compared with 220 exacerbations experienced by participants in the placebo arm.

#### Time to first moderate or severe COPD exacerbation

The time to first moderate or severe COPD exacerbation was not significantly different between astegolimab Q2W and placebo (hazard ratio, 0.89 [95% CI, 0.79-1.01]; *P* = .078; Table 2).

#### Patient-reported outcomes and lung function

There was a nominally significant increase in the proportion of participants with improvement in SGRQ-C (decrease from baseline of ≥4 points at week 52) with astegolimab Q2W vs placebo (57% vs 49%, respectively; odds ratio, 1.35 [95% CI, 1.11-1.64]; *P* = .0029; Table 2); however, no significant differences were observed for mean change from baseline in SGRQ-C, E-RS:COPD, or FEV_1_ (Table 2).

### Safety

An overview of AEs for astegolimab Q2W and placebo is shown in Table 3. Astegolimab Q2W was well tolerated with fewer AEs or serious AEs (SAEs) compared with placebo (AEs: 365.19 vs 408.39 per 100 patient-years; SAEs: 38.92 vs 47.96 per patient-years). The rate of infections was similar between treatment arms, and injection site reactions were numerically less common with astegolimab Q2W than placebo (25.02 vs 33.78 per 100 patient-years). The rate of MACEs was 2.88 and 3.74 per 100 patient-years in the astegolimab Q2W and placebo arms, respectively. Deaths occurred in 2.7% and 3.5% of participants in the astegolimab Q2W and placebo arms, respectively, and no differences or trends were observed between treatment arms in vital signs or laboratory values.

**Table 3:** Overview of treatment-emergent adverse events for astegolimab every 2 weeks vs placebo (safety evaluable patients).

Adverse event	Astegolimab Q2W	Placebo
	**(*n*** = **902)**	**(*n*** = **877)**
**Total patient-years at risk^a^**	1043.3	1015.4
**Any AE**		
** Number of AEs observed**	3810	4147
** AE rate per 100 patient-years (95% CI)**	365.19 (353.60-376.79)	408.39 (395.96-420.82)
**Any SAE**		
** Number of AEs observed**	406	487
** AE rate per 100 patient-years (95% CI)**	38.92 (35.13-42.70)	47.96 (43.70-52.22)
**AE of special interest**		
** Number of AEs observed**	110	108
** AE rate per 100 patient-years (95% CI)**	10.54 (8.57-12.51)	10.64 (8.63-12.64)
**Deaths**		
** Number of AEs observed**	24	31
** AE rate per 100 patient-years (95% CI)**	2.30 (1.38-3.22)	3.05 (1.98-4.13)
**MACEs**		
** Number of AEs observed**	30	38
** AE rate per 100 patient-years (95% CI)**	2.88 (1.85-3.90)	3.74 (2.55-4.93)
**Infections**		
** Number of AEs observed**	756	724
** AE rate per 100 patient-years (95% CI)**	72.46 (67.30-77.63)	71.30 (66.11-76.49)
**Injection site reactions**		
** Number of AEs observed**	261	343
** AE rate per 100 patient-years (95% CI)**	25.02 (21.98-28.05)	33.78 (30.20-37.35)

Abbreviations: AE, adverse event; CI, confidence interval; MACE, major adverse cardiovascular event; Q2W, every 2 weeks; SAE, serious adverse event.

a Total patient-years at risk is the sum of all patients over the time intervals (in years) from the start of study treatment to the end of the AE reporting period.

Astegolimab Q4W safety data are provided in Table S4.

## Discussion

The phase 2b ALIENTO and phase 3 ARNASA trials are the first pivotal trials to report results for anti-ST2/IL-33–targeted therapies for COPD.^14^ Both trials enrolled participants with COPD and a history of frequent exacerbations, with former/current smoking status, and enrolled irrespective of BEC or chronic bronchitis diagnosis.^14^ This pooled analysis of data from the 2 trials showed that astegolimab Q2W and Q4W significantly reduced the annualized rate of moderate or severe exacerbations vs placebo by 15% and 12%, respectively. A nominally significant treatment effect with astegolimab Q2W was also observed for annualized rate of severe exacerbations (leading to hospitalization for ≥24 hours or death) and in the responder analysis of SGRQ-C. Considering the totality of evidence from both efficacy and safety data, the astegolimab Q2W regimen appears to have a more favorable benefit/risk profile than the Q4W regimen and therefore the discussion focuses on results from the Q2W arm.

Given that every moderate or severe COPD exacerbation can negatively impact patients’ prognosis,^1^^,^^17^ the significant 15% reduction in moderate or severe exacerbations observed with astegolimab Q2W can offer clinically meaningful benefit for patients who continue to experience exacerbations while receiving optimized inhaled therapy, and who currently lack additional treatment options. Notably, participants with a baseline BEC <300 cells/µL had a reduction in moderate or severe exacerbations of 13%, while those with BEC <150 cells/µL had a reduction of 18%. These patients represent a large population with substantial unmet need as they are not eligible for any currently approved COPD biologics, which require patients to have an eosinophilic phenotype (ie, BEC ≥300 cells/µL).^1^ More broadly, efficacy with astegolimab Q2W in prespecified subgroups was generally consistent with the overall pooled population, regardless of current/former smoking status. The observation that time to first moderate or severe COPD exacerbation was not significantly different between astegolimab Q2W and placebo while the cumulative exacerbation rate was lower with astegolimab Q2W may suggest that astegolimab stabilizes exacerbation risk in patients with frequent or recurrent exacerbations. Supportive of this hypothesis, there was a suggested enhanced treatment benefit of astegolimab Q2W in those with higher burden of exacerbations (21% reduction in participants with >2 prior exacerbations). Other subgroups with possible enhanced treatment benefit of astegolimab Q2W were those with moderate airflow obstruction at baseline (GOLD 2: 27% reduction) and those without chronic bronchitis (22% reduction); however, results from unpowered subgroup analyses should be interpreted with caution.

Astegolimab Q2W reduced the total number of severe exacerbation events, with a reduction of 32% vs placebo in the annualized severe exacerbation rate. Although only nominally significant due to an early break in the statistical hierarchy, this was a similar magnitude to an exploratory analysis previously reported for mepolizumab in the MATINEE study of patients with COPD, frequent exacerbations, and baseline BEC ≥300 cells/µL.^18^ In ALIENTO and ARNASA, severe exacerbations were defined as those leading to hospitalization for >24 hours or death; the low number of deaths during the trials therefore suggests that the reduction in severe exacerbations was driven by a reduction in hospitalizations. Consequences of COPD-related hospitalizations include an increased risk of all-cause and COPD-related mortality that increases with each subsequent hospitalization.^3^ Furthermore, hospitalization for COPD is associated with decreased quality of life; patients who have a COPD-related hospitalization have worse SGRQ total score and in all domains compared with patients without exacerbations, and worse total score and impact and activity domain scores compared with patients treated as outpatients (eg, moderate exacerbation).^19^ Considering the wider societal impact of COPD, hospitalizations are the biggest contributor to COPD healthcare costs,^20^^,^^21^ and so a reduction in COPD hospitalizations of around one-third would be anticipated to have substantial economic benefits in addition to direct clinical benefits for patients.

The responder analysis of SGRQ-C suggested that an increased proportion of participants may experience a clinically meaningful improvement in quality of life (measured by improvement of ≥4 points in SGRQ-C^16^) with astegolimab Q2W vs placebo in this pooled analysis. Absolute changes in SGRQ are prone to skewing by outliers and so the responder analysis is often preferred; however, it should be acknowledged that there was no difference in total SGRQ-C score between treatment arms, and the underlying reasons for any improvement in quality of life are not yet clear. It is possible that this is a direct result of the reduction in exacerbations with astegolimab^22^^,^^23^ or that the anti-inflammatory effects of astegolimab may be improving patient symptoms during stable state. A similar improvement in SGRQ-C responder rate was observed in the pooled analysis of 2 dupilumab trials,^24^ but no ­significant difference was observed in a phase 3 trial of mepolizumab.^18^

Both doses of astegolimab were well tolerated in the pooled population, with lower rates of AEs and SAEs with astegolimab Q2W than placebo. There were very few MACEs over the 3115 patient-years of observation, with overlapping 95% CIs for the astegolimab and placebo arms. Infection rates were similar across treatment arms, indicating no detrimental impact of astegolimab on response to infection, and there were numerically fewer injection site reactions for astegolimab Q2W compared with placebo. Overall, the safety profile was generally consistent with that of previous trials.^25^

The strength of this analysis lies in the large sample size of the pooled population, with a total of 2676 participants, which provided greater statistical power than the individual trials. Data pooling was enabled by the similarity of trial designs and populations in ALIENTO and ARNASA, with the main differences being limited to upper age limit (90 vs 80 years, respectively) and the time period during which the history of prior exacerbations was considered.^14^ As a result, the significant reductions in the annualized rate of moderate or severe exacerbations for both astegolimab arms vs placebo in the pooled analysis replicated the trends of the individual trials and confirmed the consistent effect size between ALIENTO and ARNASA, despite only the Q2W arm in ALIENTO reaching significance in the individual trials.^14^ A further strength of the ALIENTO and ARNASA trials is their international nature, with participants being enrolled from 39 countries globally, including 20 countries that enrolled in both trials, with approximately 17% being of Hispanic or Latino ethnicity and approximately 21% of participants being from racial groups other than White.^14^ While this may not be truly representative of the global diversity of COPD, it is similar to other clinical trials in COPD.^18^^,^^24^^,^^26^^,^^27^ Furthermore, the individual trials enrolled a clinically heterogeneous COPD population who had a history of frequent exacerbations, with no restriction based on baseline BEC, current/former smoking status, and chronic bronchitis diagnosis. Patients with a current documented diagnosis of asthma were excluded to avoid the recruitment of patients with asthma–COPD overlap. Limitations include the observed placebo exacerbation rate (1.22) being lower than the assumed rate of 1.5, which decreased the power of the individual trials, although this effect was mitigated to some extent by pooling the data for the current analysis. The lower than expected placebo exacerbation rate may be attributed to the COVID-19 pandemic^28^^,^^29^; in a meta-analysis of the METREX and METREO trials of mepolizumab (conducted prior to the COVID-19 pandemic), the observed placebo exacerbation rate was 1.61.^30^

In conclusion, this pooled analysis of data from the ALIENTO and ARNASA trials showed that astegolimab Q2W vs placebo significantly reduced the rate of moderate or severe exacerbations by 15% over 52 weeks. Combined with a nominally significant reduction in severe exacerbations by 32% and a clinically meaningful improvement in the proportion of SGRQ-C responders, this pooled analysis suggests that targeting the ST2/IL-33 pathway may have a therapeutic role in patients with COPD and frequent exacerbations.

## Supplementary Material

aamag226_Supplementary_Data

## Data Availability

Qualified researchers may request access to individual patient-level data through the clinical trial data request platform (https://vivli.org/). Further details on Roche’s criteria for eligible studies are available here: https://vivli.org/members/ourmembers/. For further details on Roche’s Global Policy on the Sharing of Clinical Information and how to request access to related clinical trial documents, see here: https://www.roche.com/innovation/process/clinical-trials/data-sharing.
