# Supplementary material for: Astegolimab for chronic obstructive pulmonary disease with frequent exacerbations: pooled analysis of the ALIENTO and ARNASA trials
Source: Am J Respir Crit Care Med. 2026 May 18;212(9):2050–60. doi: 10.1093/ajrccm/aamag226 (PMC13519342; doi:10.1093/ajrccm/aamag226)
Supplement: Supplementary file 1 [file aamag226_supplementary_data.zip › aamag226_Supplementary_Data/Wedzicha et al_ALIENTO-ARNASA pooled manuscript_online data supplement_clean_12Aug26.pdf]

Online Data Supplement

for

Astegolimab for COPD With Frequent Exacerbations: Pooled Analysis of the ALIENTO and ARNASA Trials

Jadwiga A. Wedzicha, Àlvar Agustí, Christopher E. Brightling, Peter Calverley, James D. Chalmers, Neil J. Greening, MeiLan K. Han, Divya Mohan, Julie Ng, Alberto Papi, Nicolas Roche, Rebecca Saenz, Katerina Samara, Ruth Tal-Singer, Claus F. Vogelmeier, Xiaoying Yang, Bartolome Celli

Supplementary Methods

MACE Definition

MACE were defined as death due to cardiovascular causes, non-fatal myocardial infarction, non-fatal stroke or transient ischemic attack, unstable angina, cardiac chest pain requiring hospitalization, coronary revascularization, and congestive heart failure requiring hospitalization.

Statistical Analysis

Type 1 error was controlled using a fixed sequence testing procedure at a 5% significance level in the order of astegolimab Q2W vs. placebo then astegolimab Q4W vs. with placebo.

The adjusted annualized moderate or severe exacerbation rates (primary endpoint) and rate ratios (with 95% CIs and *P* values), subgroup analysis of the primary endpoint and annualized rate of severe exacerbations were estimated using a negative binomial regression model per ARNASA [1], with the addition of trial term to the model. A stratified log-rank test was used to compare the time to first moderate or severe exacerbations between astegolimab and placebo, per ARNASA [1], with the addition of trial term to the model. The hazard ratios for time to first moderate or severe exacerbation were estimated by a Cox proportional hazards regression model, per ARNASA [1], with the addition of trial term to the model.

A mixed model for repeated measures was used to estimate the difference in mean absolute change from baseline in SGRQ-C total score, FEV_1, and E-RS:COPD score between each astegolimab treatment arm and placebo, per ARNASA [1], with the addition of trial term to the model.

The odds ratio of having clinically meaningful improvement in SGRQ-C total score, i.e. ≥4-point decrease from baseline in SGRQ-C [2], was estimated using a logistic regression model per ARNASA [1], with the addition of trial term to the model.

Supplementary Tables and Figures

Table S1. Hierarchy of Testing for Secondary Endpoints in the ALIENTO and ARNASA Pooled Analysis

**Endpoint**
Absolute change from baseline in HRQoL at Week 52, as assessed through the SGRQ-C total score
Annualized rate of severe COPD exacerbations over the 52-week treatment period
Time to first moderate or severe COPD exacerbation during the 52-week treatment period
Absolute change from baseline in post-bronchodilator FEV_1 at Week 52
Absolute change from baseline in E-RS:COPD total score at Week 52
Proportion of participants with improvement in HRQoL, defined as a decrease from baseline of ≥4 points in SGRQ-C total score, at Week 52
Annualized rate of moderate or severe COPD exacerbations over the 52-week treatment period in participants with blood eosinophil levels <300 cells/µL

Definition of abbreviations: COPD=chronic obstructive pulmonary disease; E-RS:COPD=Evaluating Respiratory Symptoms in COPD; FEV_1=forced expiratory volume in 1 second; HRQoL=health-related quality of life; SGRQ-C=St George's Respiratory Questionnaire for COPD.

Table S2. Baseline Demographics and Characteristics (mITT Population)

	Astegolimab Q2W (*n* = 892)	Astegolimab Q4W (*n* = 896)	Placebo (*n* = 888)	All Patients (*N* = 2676)
Mean age (SD) – years	66.76 (7.93)	67.10 (7.68)	67.14 (7.27)	67.00 (7.63)
Sex – no. (%)				
Male	538 (60.3)	539 (60.2)	536 (60.4)	1613 (60.3)
Female	354 (39.7)	357 (39.8)	352 (39.6)	1063 (39.7)
Race – no. (%)				
American Indian or Alaska Native	9 (1.0)	5 (0.6)	11 (1.2)	25 (0.9)
Asian	112 (12.6)	111 (12.4)	123 (13.9)	346 (12.9)
Black or African American	29 (3.3)	46 (5.1)	35 (3.9)	110 (4.1)
Native Hawaiian or Other pacific islander	5 (0.6)	2 (0.2)	2 (0.2)	9 (0.3)
White	716 (80.3)	705 (78.7)	699 (78.7)	2120 (79.2)
Multiple	8 (0.9)	8 (0.9)	10 (1.1)	26 (1.0)
Not reported or unknown[*]	13 (1.5)	19 (2.1)	8 (0.9)	40 (1.5)
Ethnicity – no. (%)				
Hispanic or Latino	147 (16.5)	144 (16.1)	154 (17.3)	445 (16.6)
Smoking status – no. (%)				
Current smoker	302 (33.9)	305 (34.0)	303 (34.1)	910 (34.0)
Former smoker	590 (66.1)	591 (66.0)	585 (65.9)	1766 (66.0)
Blood eosinophil counts				
n	888	895	888	2671
Mean (SD), cells/µL	183.5 (159.1)	187.6 (198.6)	176.5 (116.2)	182.6 (161.6)
<150 cells/µL – no. (%)	459 (51.7)	434 (48.5)	427 (48.1)	1320 (49.4)
≥150 to <300 cells/µL – no. (%)	306 (34.5)	344 (38.4)	360 (40.5)	1010 (37.8)
≥300 cells/µL – no. (%)	123 (13.9)	117 (13.1)	101 (11.4)	341 (12.8)
FEV_1				
n	889	895	884	2668
Mean FEV_1 (SD)[†] – L	1.23 (0.48)	1.18 (0.46)	1.21 (0.50)	1.21 (0.48)
Mean %FEV_1 (SD)[†] – %	45.8 (15.2)	44.8 (15.2)	45.2 (15.6)	45.3 (15.3)
Number of COPD exacerbation within 12 months prior to screening[‡] – no. (%)				

n	891	895	886	2672
2	612 (68.7)	617 (68.9)	649 (73.3)	1878 (70.3)
>2	279 (31.3)	278 (31.1)	237 (26.7)	794 (29.7)
COPD standard-of-care at baseline[§] – no. (%)				
n	891	895	887	2673
ICS/LABA/LAMA	695 (78.0)	686 (76.6)	672 (75.8)	2053 (76.8)
ICS/LABA	128 (14.4)	130 (14.5)	133 (15.0)	391 (14.6)
LABA/LAMA	67 (7.5)	79 (8.8)	78 (8.8)	224 (8.4)
ICS	1 (0.1%)	0 (0)	2 (0.2)	3 (0.1)
ICS/LAMA	0 (0)	0 (0)	1 (0.1)	1 (<0.1)
LAMA	0 (0)	0 (0)	1 (0.1)	1 (<0.1)
Use of azithromycin[§] – no. (%)	15 (1.7)	35 (3.9)	38 (4.3)	88 (3.3)
Use of roflumilast[§] – no. (%)	35 (3.9)	17 (1.9)	26 (2.9)	78 (2.9)
Diagnosis of chronic bronchitis – no. (%)	420 (47.1)	436 (48.7)	427 (48.1)	1283 (47.9)
GOLD stage at baseline – no. (%)				
n	889	895	884	2668
GOLD 1 (mild)	9 (1.0)	8 (0.9)	11 (1.2)	28 (1.0)
GOLD 2 (moderate)	333 (37.5)	308 (34.4)	303 (34.3)	944 (35.4)
GOLD 3 (severe)	392 (44.1)	413 (46.1)	408 (46.2)	1213 (45.5)
GOLD 4 (very severe)	155 (17.4)	166 (18.5)	162 (18.3)	483 (18.1)
mMRC – no. (%)				
0	24 (2.7)	17 (1.9)	32 (3.6)	73 (2.7)
1	58 (6.5)	72 (8.0)	63 (7.1)	193 (7.2)
2	419 (47.0)	398 (44.4)	392 (44.1)	1209 (45.2)
3	372 (41.7)	382 (42.6)	378 (42.6)	1132 (42.3)
4	19 (2.1)	27 (3.0)	23 (2.6)	69 (2.6)
Mean SGRQ-C total score (SD)	59.9 (17.7) [*n* = 886]	59.7 (18.0) [*n* = 892]	59.6 (18.2) [*n* = 881]	59.8 (18.0) [*n* = 2659]
Mean E-RS:COPD total score (SD)	16.0 (6.5) [*n* = 872]	16.3 (6.9) [*n* = 871]	15.9 (6.6) [*n* = 865]	16.1 (6.7) [*n* = 2608]

[*]Unknown refers to participants for whom race is not known. Participants who refuse to answer or do not identify with any of the options listed are also counted under unknown. Not reported refers to participants for whom race cannot be collected per local laws and regulations. [†]All spirometry parameters are based on post-bronchodilator sessions. [‡]Number of COPD exacerbation within 12 months prior to screening was evaluated in a

12-month period in the 24 months prior to screening in ALIENTO. [§]Baseline use of COPD standard-of-care, azithromycin, and roflumilast is determined based on prior and concomitant drugs that were initiated before the initial study drug and were ongoing at the time of the initial study drug administration.

Definition of abbreviations: %FEV_1=percent predicted forced expiratory volume in 1 second; COPD=chronic obstructive pulmonary disease; E-RS:COPD=Evaluating Respiratory Symptoms in COPD; FEV_1=forced expiratory volume in 1 second; GOLD=Global Initiative for Chronic Obstructive Lung Disease; ICS=inhaled corticosteroid; LABA=long-acting beta agonist; LAMA=long-acting muscarinic antagonist; mITT=modified intent-to-treat; mMRC=modified Medical Research Council; Q2W=every 2 weeks; Q4W=every 4 weeks; SD=standard deviation; SGRQ-C=St George's Respiratory Questionnaire for COPD.

Table S3. Secondary Efficacy Endpoints for Astegolimab Q4W vs. Placebo (mITT Population)

	**Astegolimab Q4W (*n* = 896)**	**Placebo (*n* = 888)**
Absolute change from baseline in SGRQ-C at Week 52		
Adjusted mean (SE)	–6.95 (0.59) [*n* = 760]	–5.80 (0.59) [*n* = 741]
Difference in adjusted mean vs. placebo (95% CI)	–1.15 (–2.78 to 0.49); *P*=0.1693	
Annualized rate of severe COPD exacerbations over the 52-week treatment period		
Total number of severe exacerbation events	178	220
Adjusted rate	0.17	0.22
Adjusted rate ratio vs. placebo (95% CI)	0.79 (0.62–1.02); *P*=0.0663	
Time to first moderate or severe COPD exacerbation during the 52-week treatment period		
Median (95% CI), weeks	43.0 (38.1–48.0)	36.3 (32.9–44.4)
Stratified hazard ratio vs. placebo (95% CI)	0.92 (0.81–1.04); *P*=0.1977	
Absolute change from baseline in post-bronchodilator FEV_1 at Week 52		
Adjusted mean (SE), mL	–13.37 (8.04) [*n* = 727]	–19.48 (8.12) [*n* = 710]
Difference in adjusted mean vs. placebo (95% CI)	6.11 (–16.30 to 28.52); *P*=0.5930	
Absolute change from baseline in E-RS:COPD total score at Week 52		
Adjusted mean (SE)	–1.61 (0.20) [*n* = 711]	–1.68 (0.21) [*n* = 697]
Difference in adjusted mean vs. placebo (95% CI)	0.07 (–0.50 to 0.64); *P*=0.8106	
Proportion of patients with decrease from baseline of ≥4 points in SGRQ-C at Week 52		
Adjusted responder rate, %	53.6 [*n* = 872]	49.2 [*n* = 845]
Odds ratio vs. placebo (95% CI)	1.19 (0.98–1.45); *P*=0.0770	
Annualized rate of moderate or severe COPD exacerbations over the 52-week treatment period in patients with baseline eosinophil count <300 cells/µL		
Adjusted rate	1.08 [*n* = 778]	1.19 [*n* = 787]
Adjusted rate ratio vs. placebo (95% CI)	0.90 (0.80–1.02); *P*=0.0962	

Endpoints listed in order of fixed sequence testing procedures.

Definition of abbreviations: CI=confidence interval; COPD=chronic obstructive pulmonary disease; E-RS:COPD=Evaluating Respiratory Symptoms in COPD; FEV_1=forced expiratory volume in 1 second; mITT=modified intent-to-treat; Q4W=every 4 weeks; SE=standard error; SGRQ-C=St George's Respiratory Questionnaire for COPD.

Table S4: Overview of Treatment-Emergent Adverse Events (Safety Evaluable Patients)

	**Astegolimab Q2W (*n* = 902)**	**Astegolimab Q4W (*n* = 897)**	**Placebo (*n* = 877)**	**All patients (*N* = 2676)**
Total patient-years at risk	1043.3	1056.7	1015.4	3115.4
Any adverse event				
Number of adverse events observed	3810	4258	4147	12215
Adverse event rate per 100 patient-years (95% CI)	365.19 (353.60–376.79)	402.96 (390.86–415.07)	408.39 (395.96–420.82)	392.08 (385.13–399.04)
Any serious adverse event				
Number of adverse events observed	406	438	487	1331
Adverse event rate per 100 patient-years (95% CI)	38.92 (35.13–42.70)	41.45 (37.57–45.33)	47.96 (43.70–52.22)	42.72 (40.43–45.02)
Adverse event of special interest				
Number of adverse events observed	110	89	108	307
Adverse event rate per 100 patient-years (95% CI)	10.54 (8.57–12.51)	8.42 (6.67–10.17)	10.64 (8.63–12.64)	9.85 (8.75–10.96)
Deaths				
Number of adverse events observed	24	29	31	84
Adverse event rate per 100 patient-years (95% CI)	2.30 (1.38–3.22)	2.74 (1.75–3.74)	3.05 (1.98–4.13)	2.70 (2.12–3.27)
MACE				
Number of adverse events observed	30	31	38	99
Adverse event rate per 100 patient-years (95% CI)	2.88 (1.85–3.90)	2.93 (1.90–3.97)	3.74 (2.55–4.93)	3.18 (2.55–3.80)
Infections				
Number of adverse events observed	756	796	724	2276
Adverse event rate per 100 patient-years (95% CI)	72.46 (67.30–77.63)	75.33 (70.10–80.56)	71.30 (66.11–76.49)	73.06 (70.06–76.06)

Injection site reactions				
Number of adverse events observed	261	389	343	993
Adverse event rate per 100 patient-years (95% CI)	25.02 (21.98–28.05)	36.81 (33.16–40.47)	33.78 (30.20–37.35)	31.87 (29.89–33.86)

Total patient-years at risk is the sum of all patients over the time intervals (in years) from the start of study treatment to the end of the adverse event reporting period.

Definition of abbreviations: CI=confidence interval; MACE=major adverse cardiovascular events; Q2W=every 2 weeks; Q4W=every 4 weeks.

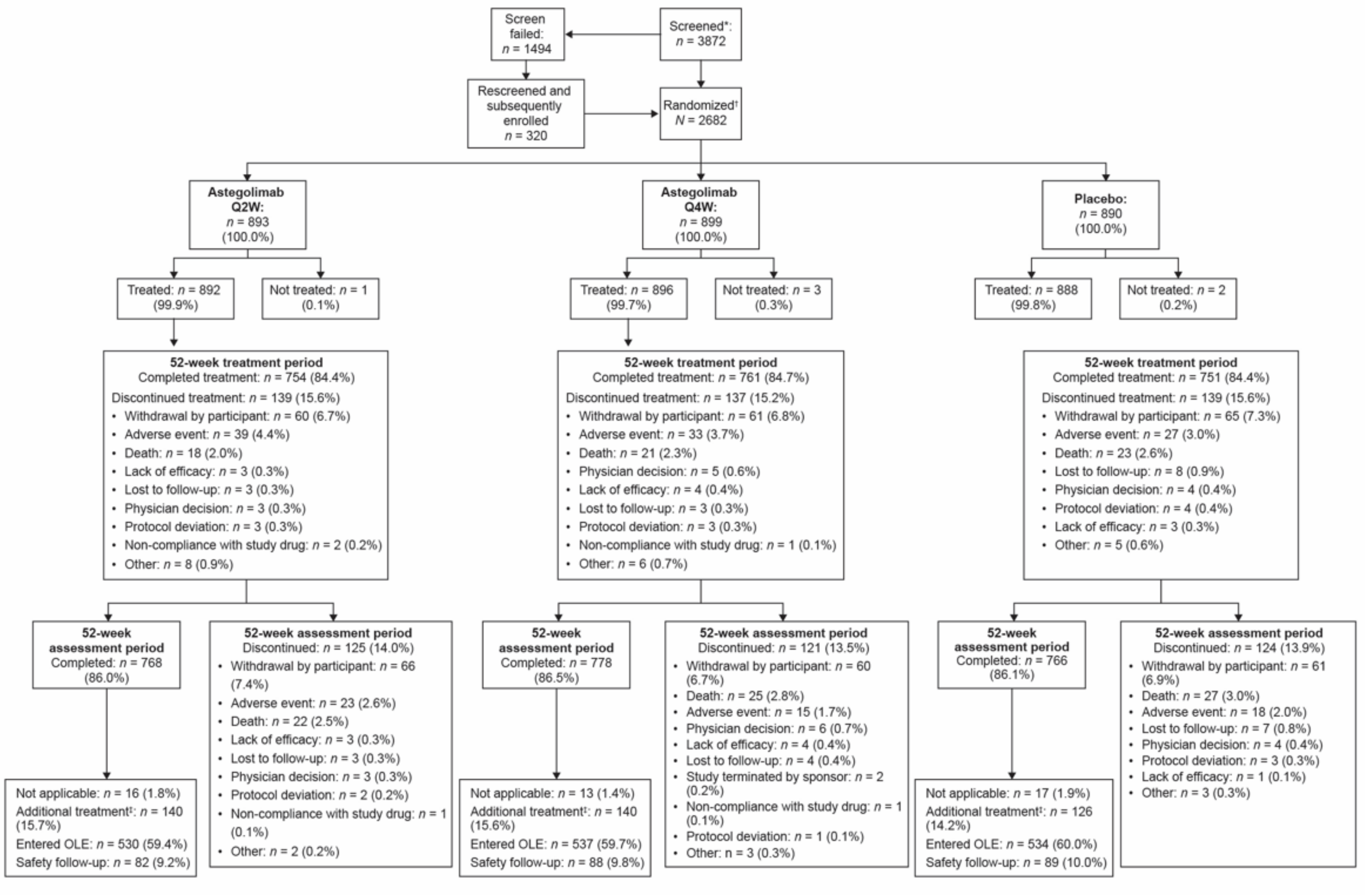

Figure S1. CONSORT diagram

*In ARNASA, 14 patients were removed due to IxRS entry errors (*n* = 10) or input of patients from an extension cohort (*n* = 4). †In ARNASA, two additional participants were excluded from analysis as they were found to be the same individual. ‡Week 88 (ALIENTO only).

Definition of abbreviations: OLE=open-label extension; Q2W=every 2 weeks; Q4W=every 4 weeks.

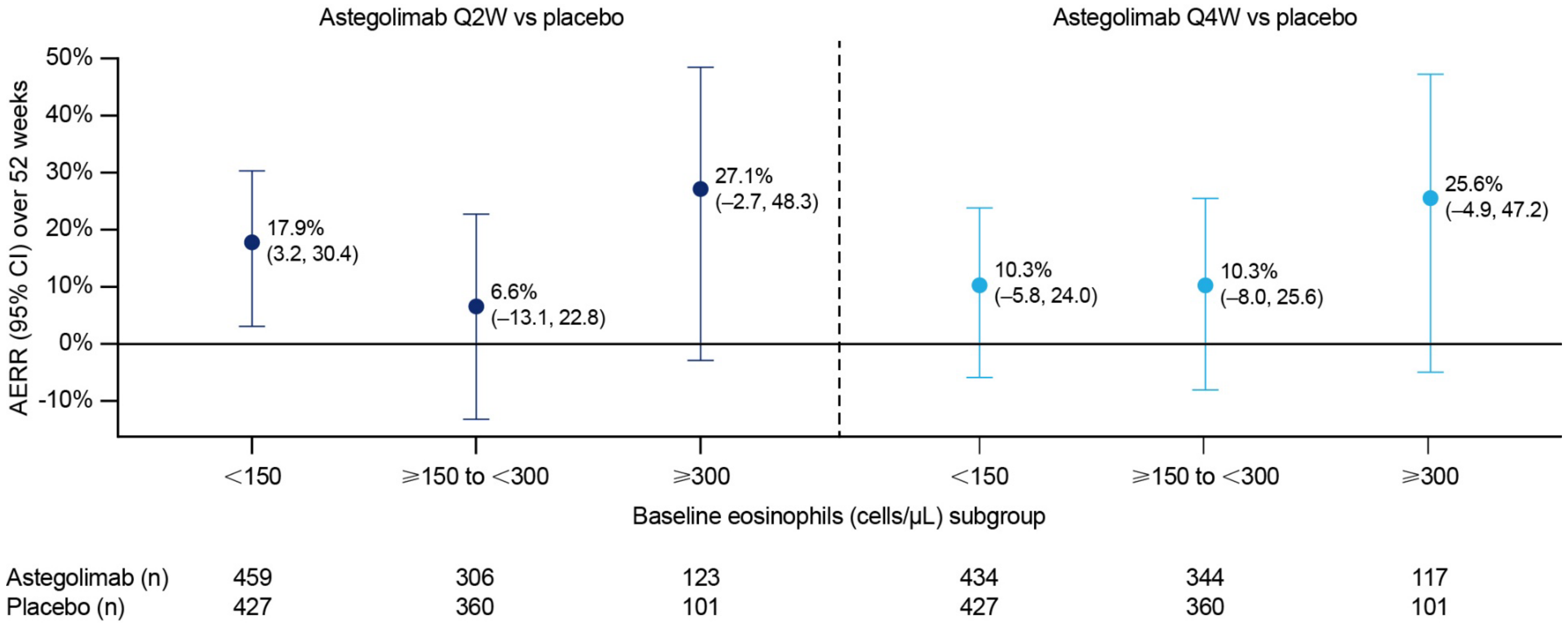

Figure S2. Annualized exacerbation rate reduction for astegolimab Q2W and Q4W vs. placebo, by baseline eosinophil groups (mITT population)

Definition of abbreviations: AERR=annualized exacerbation rate reduction; CI=confidence interval; Q2W=every 2 weeks; Q4W=every 4 weeks.

Baseline characteristic	Astegolimab Q4W *N*	Placebo *N*	RR	CI	*P*-value
All patients	896	888	0.88	(0.78–0.98)	0.0265
Baseline eosinophil level					
<150 cells/µL	434	427	0.90	(0.76–1.06)	0.1958
⩾150 cells/µL	461	461	0.86	(0.73–1.01)	0.0644
<300 cells/µL	778	787	0.90	(0.80–1.02)	0.0962
⩾300 cells/µL	117	101	0.74	(0.53–1.05)	0.0921
Age category					
40–64 years	312	301	0.86	(0.70–1.05)	0.1459
⩾65 years	584	587	0.88	(0.77–1.02)	0.0853
Sex					
Female	357	352	0.82	(0.70–0.98)	0.0266
Male	539	536	0.93	(0.79–1.08)	0.3377
Smoking status					
Current smoker	305	303	1.00	(0.82–1.22)	0.9927
Former smoker	591	585	0.82	(0.71–0.94)	0.0056
Number of COPD exacerbations*					
2	617	649	0.92	(0.79–1.07)	0.2846
>2	278	237	0.79	(0.67–0.94)	0.0070
Hospitalization for COPD†					
Yes	218	197	0.76	(0.61–0.95)	0.0150
No	678	691	0.92	(0.80–1.05)	0.2195
Baseline COPD triple therapy					
Yes	686	672	0.85	(0.75–0.96)	0.0112
No	209	215	1.01	(0.76–1.33)	0.9701
Baseline chronic bronchitis					
Yes	436	427	0.92	(0.78–1.09)	0.3556
No	460	461	0.84	(0.71–0.99)	0.0336
Baseline GOLD Stage					
Moderate	308	303	0.78	(0.62–0.99)	0.0453
Severe	413	408	0.90	(0.77–1.06)	0.2239
Very severe	166	162	0.90	(0.73–1.11)	0.3163

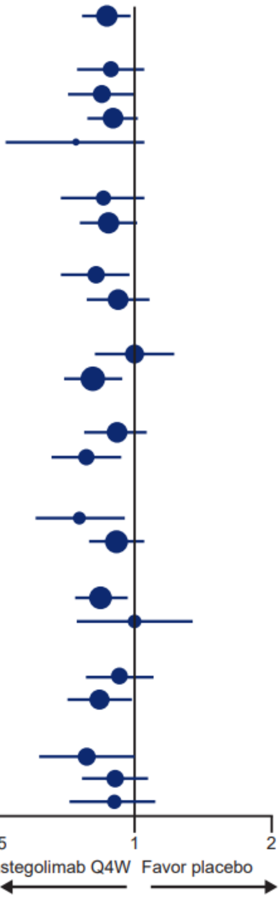

Figure S3. Adjusted annualized rate of moderate or severe COPD exacerbations for astegolimab Q4W vs. placebo, by selected prespecified subgroups (mITT population)

*For ALIENTO, the number of COPD exacerbations was based on those occurring in a 12-month period within the 24 months prior to screening. For ARNASA, it was based on 12 months prior to screening. †Hospitalization for COPD for >24 hours within the last 12 months prior to screening

Definition of abbreviations: CI=confidence interval; COPD=chronic obstructive pulmonary disease; GOLD=Global Initiative for Chronic Obstructive Lung disease; mITT=modified intent-to-treat; Q4W=every 4 weeks; RR=rate ratio.

1. Papi A, Greening NJ, Agustí À, Bhatt SP, Bremner P, Celli B*, et al.* Two randomised trials of astegolimab for COPD with frequent exacerbations, regardless of baseline blood eosinophil counts. *The Lancet* In press.

2. Jones PW. St. George's Respiratory Questionnaire: MCID. *COPD* 2005;2:75-79.
